# Targeting Prostate Cancer Cells Using Anti-Sortilin and Anti-Syndecan-1 Antibody Drug Conjugates

**DOI:** 10.3390/ijms262211145

**Published:** 2025-11-18

**Authors:** Ka Lok Li, Shane M. Hickey, Hugo Albrecht, Jessica M. Logan, Joanna Lazniewska, Courtney R. Moore, Robert D. Brooks, Ian R. D. Johnson, John J. O’Leary, Douglas A. Brooks

**Affiliations:** 1 Clinical and Health Sciences, University of South Australia, Adelaide, SA 5000, Australiajessica.logan@unisa.edu.au (J.M.L.);; 2 Department of Histopathology, Trinity College Dublin, D02 PN40 Dublin, Ireland

**Keywords:** prostate cancer, monoclonal antibodies, antibody drug conjugates, therapeutics, advanced cancer

## Abstract

Prostate cancer tissue usually involves either well formed glands, poorly formed glands or a combination of the two morphologies, which can be correlated with metabolic differences and tumor heterogeneity. This is particularly important for metastatic castration-resistant prostate cancer, where the heterogeneity and metabolic changes drive cancer progression and treatment refractory properties. Sortilin and syndecan-1 expression accurately define the two different morphologies in prostate cancer tissue, are critical to the process of metabolic regulation, and exhibit mechanistic/functional interactions during prostate cancer progression. As trans-membrane proteins that recycle from endocytic compartments to the cell surface, sortilin and syndecan-1 are attractive targets for therapeutic intervention that address the two major forms of prostate cancer. In this study, we describe an antibody-drug conjugate (ADC) strategy that utilizes monoclonal antibodies which bind to specific extracellular domains of these integral membrane proteins to elicit anticancer activity in prostate cancer cell lines. Anti-sortilin (clone 11H8) and anti-syndecan-1 (clone 6D11) monoclonal antibodies demonstrated high specificity for epitopes on the extracellular, N-terminal domains of these respective proteins and were effectively internalized into prostate cancer cell endocytic compartments. Monomethyl aurastatin E (MMAE)-conjugated ADCs exhibited low nanomolar cytotoxicity in LNCaP and PC-3 prostate cancer cells. Mechanistically, 11H8-MMAE and 6D11-MMAE triggered cytotoxicity and morphological alterations in androgen-sensitive and androgen-insensitive cells. However, the uptake of fluorescent labelled 11H8 and 6D11 antibodies appeared to be high, whereas the killing capacity of the MMAE-conjugated antibodies was less impressive, suggesting the need for further ADC development. These promising proof-of-concept ADCs are designed to exploit molecular and metabolic vulnerabilities in prostate cancer and may have utility for overcoming treatment resistance by simultaneously targeting different forms of the cancer.

## 1. Introduction

Prostate cancer remains a major global health challenge as the second most frequently diagnosed malignancy in men and a leading cause of cancer-related mortality, which accounts for over 370,000 deaths annually worldwide [1,2]. Prostate tumors exhibit a spectrum of morphological patterns, including well and poorly formed glands, cribriform structures and single-cell infiltrates [3,4,5]. This morphological variability is paralleled by metabolic reprogramming, which is increasingly recognized as a hallmark of prostate cancer progression and therapeutic resistance [6]. This interplay between morphological heterogeneity and metabolic plasticity not only underpins disease progression and therapeutic resistance but also presents significant challenges for the identification of robust, actionable targets for antibody-based therapies [7,8]. With the incidence of prostate cancer expected to double by 2040, there is an unmet need to develop new precision therapies to combat this prevalent disease [9,10]. Therapeutic strategies for prostate cancer are typically stratified according to disease morphology and clinical presentation. For localized low-grade disease (well formed gland morphology), radical prostatectomy and radiotherapy remain the mainstays of curative intent [7,8]. While these interventions decrease the risk of disease progression and metastasis compared to active surveillance, they can be associated with life-altering adverse effects, including urinary, bowel, and sexual dysfunction [11,12]. Patients with advanced (poorly formed gland morphology) or recurrent disease typically undergo androgen deprivation therapy (ADT) to suppress the tumor growth [13,14]. Unfortunately, within 18 months to 3 years of initiating ADT, treatment resistance inevitably emerges, resulting in the development of castration-resistant prostate cancer (CRPC), a lethal phenotype characterized by the activation of evasive signaling pathways that drive tumor growth and survival [7,8,14]. Treatment of CRPC is limited to taxane-based chemotherapy (e.g., docetaxel, cabazitaxel) and next-generation androgen receptor (AR) pathway inhibitors (e.g., enzalutamide, abiraterone); however, durable remissions remain elusive, and the use of these drugs causes significant off-target toxicities [13].

In recent years, antibody-based therapeutics have garnered considerable interest as tumor-selective targeting agents that enhance therapeutic indices [15]. Monoclonal antibodies, by virtue of their high specificity for antigens, have been developed as vehicles for the targeted delivery of cytotoxic payloads (such as chemotherapeutic agents or radionuclides), resulting in the generation of antibody-drug conjugates (ADCs) and antibody-radionuclide conjugates [16,17,18]. Clinical studies show that antibody targeting can enhance therapeutic efficacy and improve prostate cancer patient survival [19]. Despite the great potential offered by ADC therapeutics, the bottleneck for this approach is the identification of suitable tumor-specific antigens.

Antibody-based prostate cancer therapies currently rely on a limited repertoire of targets, including prostate-specific membrane antigen (PSMA) and prostate stem cell antigen (PSCA). PSMA is a type II transmembrane glycoprotein that is overexpressed in prostate cancer and is associated with poor prognosis and the development of CRPC [20,21]. Targeting PSMA using ADCs and radionuclide conjugates has shown promise in clinical trials [22], but PSMA is not uniformly or universally expressed in all prostate cancers [23,24]. Moreover, PSMA is expressed in a range of benign and malignant non-prostatic tissues that likely limits the selectivity and safety of PSMA-targeted therapies [23]. Similarly, PSCA is upregulated in many prostate cancers and correlates with higher tumor grade and poor prognosis, but its expression is not restricted to the prostate [25,26]. Thus, while these targets have enabled the development of antibody-based therapeutics, variable expression and limitations for effectively treating advanced disease highlight the need for novel biomarkers that are more uniformly expressed and more closely linked to disease pathogenesis.

Emerging evidence indicates that cell surface proteins involved in metabolic regulation and cell signaling may serve as promising biomarkers and therapeutic targets for prostate cancer [6]. For example, sortilin and syndecan-1 have attracted attention for their high tumor sensitivity/specificity and roles in modulating glucose and lipid metabolism, cell proliferation, and tumor progression [4,27,28]. Sortilin, a transmembrane sorting receptor, is implicated in the regulation of glucose transporter type 4 (GLUT4) trafficking and lipoprotein lipase (LPL) degradation, as well as in the secretion of neural apoptosis-regulated convertase 1 (NARC-1/PCSK9), which influences low-density lipoprotein receptor (LDLR) turnover and cholesterol homeostasis [27,29,30,31]. Increased sortilin expression is observed in low-grade prostate cancer and has a potential role in the metabolic programming and aggressive behavior of tumor cells [4,27,28]. In low-grade disease, sortilin mediates endocytosis and degradation of progranulin, a growth factor that promotes proliferation, metastasis, and resistance to therapy [32,33]. Loss of sortilin expression in high-grade or CRPC may lead to increased extracellular progranulin, decreased NARC-1, and a shift in lipid metabolism, further driving disease aggressiveness [27]. Syndecan-1, a heparan sulfate proteoglycan, is another cell surface protein implicated in the regulation of lipid metabolism and cancer progression. Syndecan-1 modulates interactions with the extracellular matrix, growth factor signaling, and cellular adhesion, and its expression is associated with more aggressive disease phenotypes and poorer prognosis in prostate cancer [4,27,28]. The reciprocal interconnected regulation of sortilin and syndecan-1 may contribute to the regulation of metabolic and morphological heterogeneity observed in prostate cancer [27]. The dual targeting of sortilin and syndecan-1 may offer specific advantages over existing therapeutic interventions. For example, the combinatorial use of sortilin and syndecan-1 may prevent prostate cancer from transitioning between different forms, limit metabolic reprogramming, and restrict the cancer refractory potential. The high sensitivity and specificity of the sortilin and syndecan-1 biomarkers offer a significant advantage over PSMA, as the latter has variable expression within cancer tissue and is not overexpressed in all prostate cancers [34]. The novel therapeutic approach of simultaneously targeting the two main forms of prostate cancer may also provide a solution to the development of therapeutic resistance.

## 2. Results

### 2.1. Characterization of Anti-Sortilin Monoclonal Antibody

**Figure 1:**
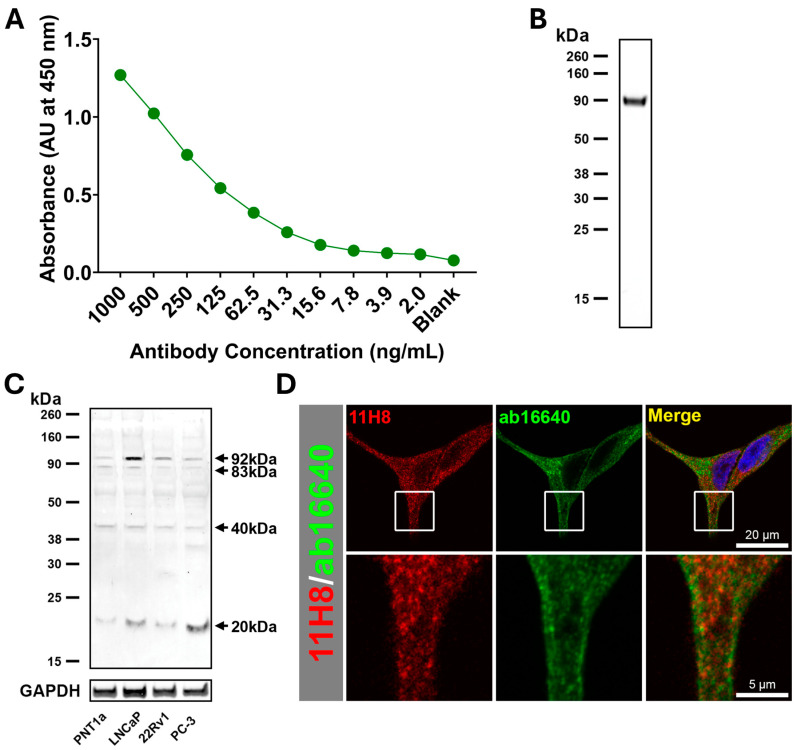
Characterization of anti-sortilin monoclonal antibody 11H8 specificity. (**A**) Indirect ELISA of anti-sortilin monoclonal antibody 11H8 against recombinant protein (100 ng/well). (**B**) Western blot of 11H8 monoclonal antibody against 200 ng of sortilin recombinant protein (90 kDa form lacking membrane spanning domain). (**C**) Western blot of 11H8 against 10 μg of total protein cell extract from either PNT1a, LNCaP, 22Rv1 or PC-3 cell lines compared to GAPDH controls. (**D**) Immunofluorescence of 11H8 monoclonal antibody (red) compared to commercial polyclonal antibody ab16640 (green), showing colocalization (yellow), and counterstained with DAPI (blue); fluorescence data representative of triplicate experiments; scale bars are 20 μm for the top row and 5 μm for the bottom row.

The monoclonal antibody 11H8 (IgG2b) was generated against a specific peptide motif, LANNTHQHVFDDLRG, contained in the sortilin protein sequence. Genscript adhered to ISO 9001 standard reagent production with full independent peptide and protein ELISA specificity and Western blotting defined against peptide and recombinant protein (representative ELISA and Western blotting against recombinant protein in Figure 1A,B, respectively). A BLAST-search (BLAST+ 2.14.0) analysis based on a six-amino acid sequence identity comparison was conducted to identify any proteins with a common sequence that may potentially interact with the anti-sortilin antibody generated against this peptide, but none were identified. On prostate cell extracts, the monoclonal antibody 11H8 detected proteins at approximately 92, 83, 40 and 20 kDa, and there was qualitative evidence of higher expression of the 92 kDa and 20 kDa molecular forms in prostate cancer cells (LNCaP, 22RV1 and PC-3) compared to non-malignant cell extracts (PNT1a) (Figure 1C). Immunofluorescence colocalization was performed with Alexa Fluor 647-conjugated 11H8 monoclonal antibody and compared to a knockout validated commercial sortilin polyclonal antibody (ab16640) using a secondary Donkey anti-Rabbit IgG Alexa Fluor 488 conjugate (Figure 1D). The antibody 11H8 exhibited a pattern of punctate labelling, and spatial correlation analysis revealing that there was moderate colocalization with the commercial sortilin polyclonal antibody, as evidenced by an average Pearson correlation coefficient of 0.69.

### 2.2. Anti-Sortilin Antibody Uptake and Internalization into Non-Malignant and Malignant Prostate Cells

**Figure 2:**
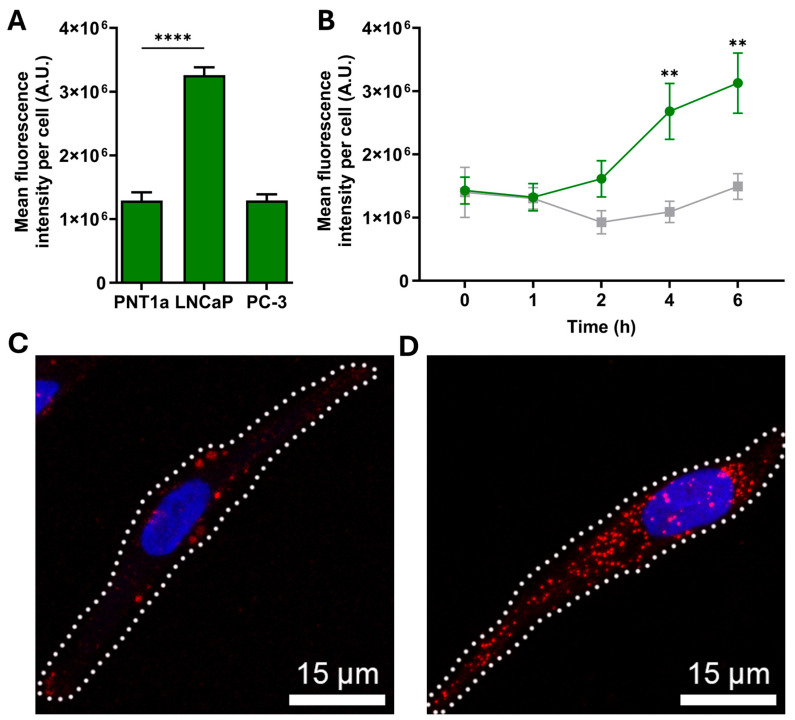
Cellular uptake and internalization of anti-sortilin monoclonal antibody 11H8. (**A**) Alexa Fluor^®^ 647-conjugated anti-sortilin monoclonal antibody 11H8 internalization by live PNT1a non-malignant and LNCaP and PC-3 malignant prostate cell lines after 6 h (one-way ANOVA using Dunnett’s multiple comparison *n* = 3, **** *p* < 0.0001; error bars represent the SEM). (**B**) Alexa Fluor^®^ 647-conjugated 11H8 (green dots) or IgG2b isotype control (ab18421; grey dots) internalization by live LNCaP cells at 1, 2, 4, and 6 h. Antibody internalization was measured as mean fluorescence intensity and represents three individual experiments (unpaired *t*-test, ** *p* < 0.01; error bars represent the SEM). (**C**,**D**) Representative maximum-intensity projections of confocal micrographs of sortilin antibody uptake in LNCaP cells captured using live cells (**C**) or fixed permeabilized cells (**D**) labelled with Alexa Fluor^®^ 647-conjugated 11H8 antibody (red) and Hoechst 33,342 to counterstain the cell nuclei (blue).

**Figure 3:**
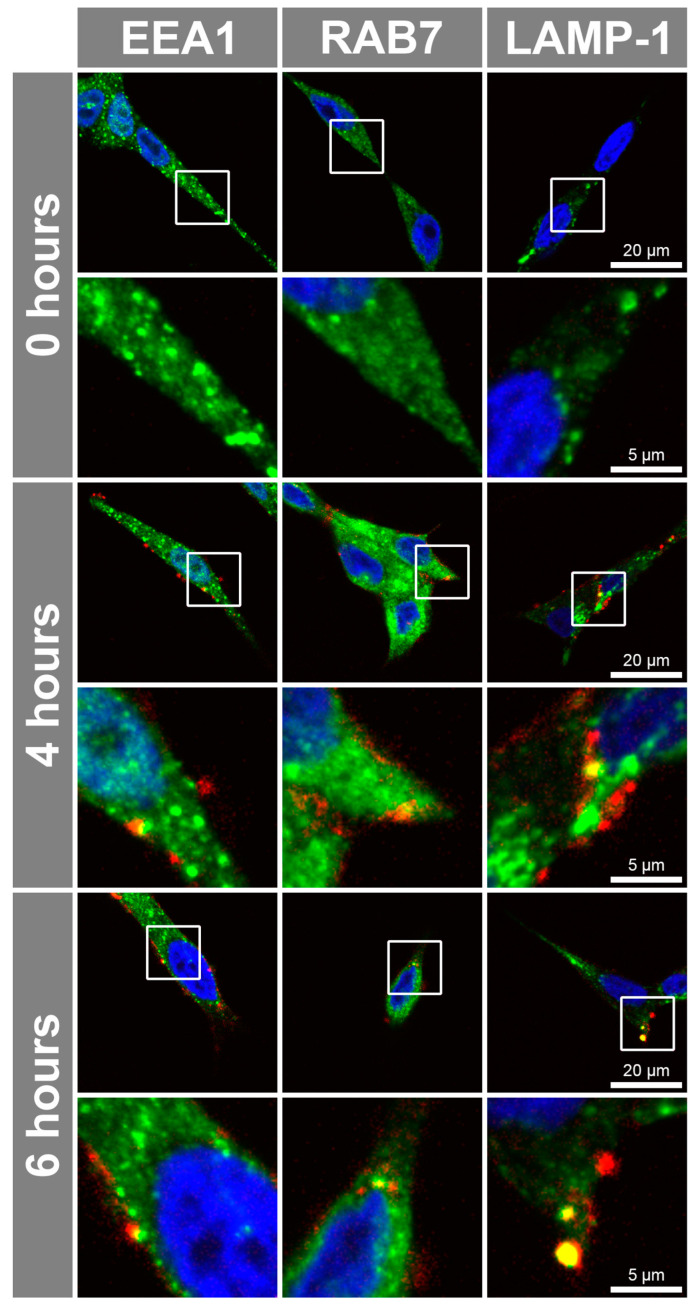
Endosome-lysosome uptake of anti-sortilin monoclonal antibody 11H8. Alexa Fluor^®^ 647-conjugated 11H8 monoclonal antibody uptake in LNCaP cells at 0, 4 and 6 h with EEA1, Rab7 and LAMP-1 labelled endosomes-lysosomes (green), 11H8 (red) and nuclei (blue), with compartment colocalization (yellow). A cropped and 4× enlarged area (below) is indicated by white box. Representative confocal micrographs from *n* = 5 replicates.

Antibody internalization via endocytosis is a crucial aspect for the development of antibody therapeutics, as it ensures the effective delivery of antibody to its intracellular target [35]. To investigate antibody internalization via cell surface receptors, live prostate cells were incubated with Alexa Fluor^®^ 647-conjugated sortilin monoclonal antibody. There was a significant increase in the 11H8 monoclonal antibody fluorescence in LNCaP compared to both PNT1a and PC-3 cells, following a 6 h incubation (Figure 2A). To confirm the specificity and time course for the internalization of the 11H8 monoclonal antibody, LNCaP cells were incubated with either Alexa Fluor^®^ 647-conjugated 11H8 monoclonal antibody or an isotype control, over a 6 h time course (Figure 2B). The endocytosis of 11H8-bound sortilin exhibited a progressive increase in intensity, with a significant increase in fluorescence signal observed after 4 and 6 h incubation, compared to the isotype control that did not show any significant uptake at these time points (*p* < 0.01; Figure 2B). To assess the uptake capacity via cell surface receptors, live LNCaP cells were incubated with the 11H8 monoclonal antibody and compared to fixed permeabilized cells. Live cell uptake revealed similar punctate labelling but with reduced numbers of vesicles in the live cell experiments (Figure 2C) compared to the fixed/permeabilized cells (Figure 2D), indicating a possible difference between cell surface availability of sortilin versus the total intracellular pool. There was evidence of Alexa Fluor^®^ 647-conjugated 11H8 monoclonal antibody delivery to EEA1 early endosomes, Rab7 late endosomes, and LAMP-1 endosomes-lysosomes after 4–6 h incubation on live LNCaP cells, indicating delivery to endocytic compartments (Figure 3). However, there were compartments that appeared to be labelled with Alexa Fluor^®^ 647-conjugated 11H8 monoclonal antibody, but not co-locating with EEA1, Rab7 and LAMP-1, suggesting delivery to other endosome-lysosome compartments.

### 2.3. Characterization of Anti-Syndecan-1 Monoclonal Antibody

**Figure 4:**
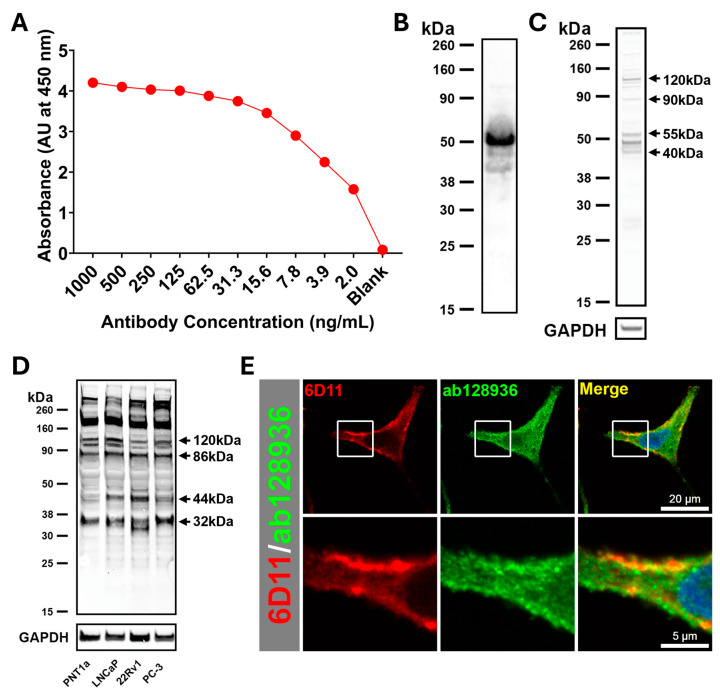
Characterization of anti-syndecan-1 monoclonal antibody 6D11 specificity. (**A**) Indirect ELISA of anti-syndecan-1 monoclonal antibody 6D11 against recombinant protein (100 ng/well). (**B**) Western blot of 6D11 against 200 ng of syndecan-1 recombinant protein. (**C**) Western blot of 6D11 against 10 μg of total protein cell extract from HeLa cells (**D**) Western blot of 6D11 against 10 μg of total protein cell extract from either PNT1a, LNCaP, 22Rv1 or PC-3 cell lines compared to GAPDH controls. (**E**) Immunofluorescence of 6D11 monoclonal antibody (red) compared to commercial syndecan-1 polyclonal antibody ab128936 (green), showing colocalization (yellow), and counterstained with DAPI (blue); fluorescence data representative of triplicate experiments; scale bars are 20 μm for the top row and 5 μm for the bottom row.

The monoclonal antibody 6D11 (IgG2b) was generated against a specific peptide sequence, AREQEATPRPRETTQLPTTH. Genscript adhered to ISO 9001 standard reagent production with full independent peptide and protein ELISA specificity and Western blotting defined against peptide and recombinant protein (representative ELISA and Western blotting against recombinant protein in Figure 4A and Figure 4B, respectively). A BLAST-search analysis based on a six-amino acid sequence identity comparison was conducted to identify any proteins with a common sequence that may potentially interact with anti-syndecan-1 antibody generated against this peptide. Potential sequence similarity with plasma protease C1 inhibitor (C1Inh; QPTQPTTQLPTDSPT) was identified, and some residual activity was detected against this protein (AU at 450 nm of 0.367 C1Inh compared to 3.252 for syndecan-1 using full-length recombinant proteins and ~8 ng/mL of each antibody; full ELISA). A Western blot against HeLa cell extracts produced a set of molecular forms, including those at 40–55 kDa, 90 kDa and 120 kDa (Figure 4C). In prostate cell extracts, a more complicated pattern was detected, consistent with extensive syndecan-1 processing and glycolytic modification. The monoclonal antibody 6D11 detected molecular forms at approximately 32–36, 44, 70, 86, 100, 120, 175, and 260 kDa, along with other less-intense molecular forms between 45–80 kDa and below 30 kDa (Figure 4D). In particular, the monoclonal antibody 6D11 detected 32 kDa and 70 kDa molecular forms, which corresponded to the known monomeric and dimeric forms of syndecan-1, respectively [4,36,37]. There was evidence of more of the 44 kDa molecular form in the malignant prostate cancer cells compared to the non-malignant cells, but very similar patterns overall (Figure 4D). The monoclonal antibody 6D11 displayed a very specific pattern of detection by immunofluorescence with minimal detection of the intracellular organelles by the syndecan-1 polyclonal antibody ab128936. Specifically, the monoclonal antibody 6D11 detected organelles consistent with endosomes in close proximity to the cell surface and co-labelling with the syndecan-1 polyclonal antibody ab128936 (Figure 4E; Pearson correlation coefficient of 0.82 and a Manders Overlap Coefficient of 0.48).

A second monoclonal antibody, 10A3, was generated against an intracellular cytoplasmic domain epitope on syndecan-1 (IgG2b antibody against the peptide sequence EPKQANGGAYQKPTK) and used as a negative control for uptake studies (i.e., intracellular epitope not exposed at the cell surface).

### 2.4. Anti-Syndecan-1 Antibody Uptake and Internalization into Non-Malignant and Malignant Prostate Cells

**Figure 5:**
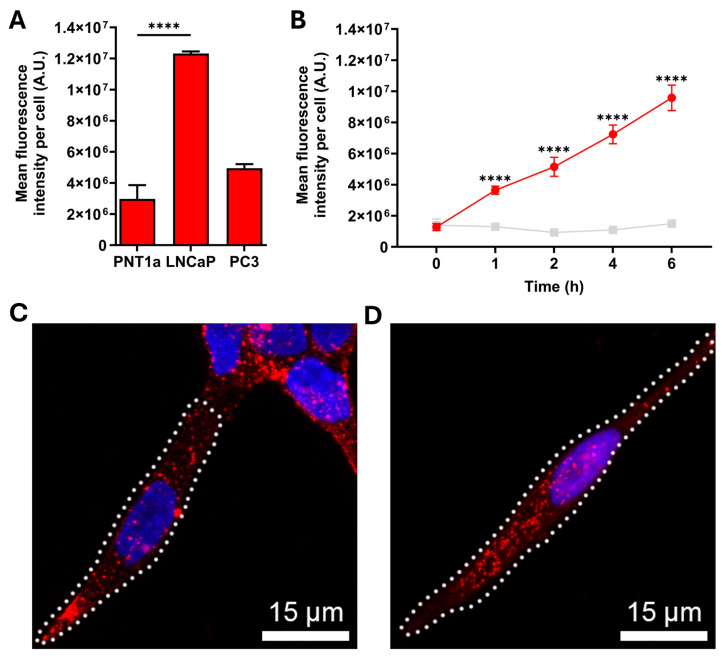
Cellular uptake and internalization of anti-syndecan-1 monoclonal antibody 6D11. (**A**) Alexa Fluor^®^ 647-conjugated anti-syndecan-1 monoclonal antibody 6D11 internalization by PNT1a non-malignant and LNCaP and PC-3 malignant prostate cell lines after 6 h (one-way ANOVA using Dunnett’s multiple comparison *n* = 3, **** *p* < 0.0001; error bars represent the SEM). (**B**) Alexa Fluor^®^ 647-conjugated 6D11 (red dots) or IgG2b isotype control (ab18421; grey dots) internalization by LNCaP cells at 1, 2, 4, and 6 h. Antibody internalization was measured as mean fluorescence intensity and represents three individual experiments (unpaired *t*-test, **** *p* < 0.0001; error bars represent the SEM). (**C**,**D**) Representative maximum-intensity projections of confocal micrographs of sortilin antibody uptake in LNCaP cells captured using live cells (**C**) or fixed and permeabilized cells (**D**) labelled with Alexa Fluor^®^ 647-conjugated 6D11 antibody (red) and Hoechst 33,342 to counterstain the cell nuclei (blue).

**Figure 6:**
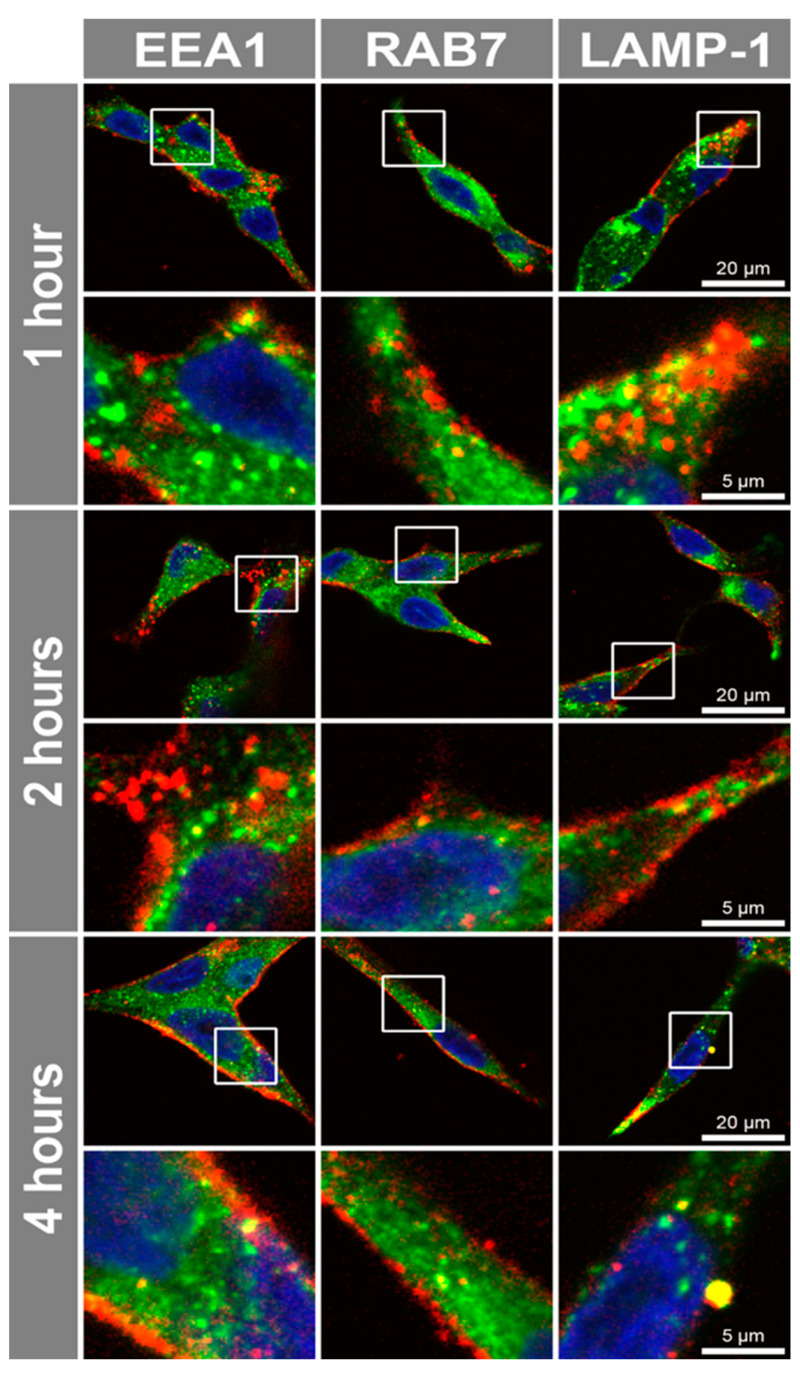
Endosome-lysosome uptake of anti-syndecan-1 monoclonal antibody 6D11. Alexa Fluor^®^ 647-conjugated anti-syndecan-1 monoclonal antibody 6D11 uptake in LNCaP cells at 1, 2 and 4 h with EEA1, Rab7 and LAMP-1 labelled endosomes-lysosomes (green), 11H8 (red) and nuclei (blue), with compartment colocalization (yellow). See Figure 3 for representative 0 h controls for EEA1, Rab7 and LAMP-1 (N.B. datasets were from the same experiments for comparisons of sortilin and syndecan-1 antibody uptake). A cropped and 4× enlarged area (below) is indicated by white box. Representative confocal micrographs from *n* = 5 replicates.

To investigate antibody uptake and internalization, prostate cells were incubated with Alexa Fluor^®^ 647-conjugated anti-syndecan-1 6D11 monoclonal antibody. There was a significant increase in the 6D11 monoclonal antibody uptake in LNCaP cells, as measured by fluorescence after a 6 h incubation and compared to either PNT1a or PC-3 live cells (Figure 5A). To confirm the specificity and time course for the internalization, LNCaP cells were incubated with either Alexa Fluor^®^ 647-conjugated 6D11 monoclonal antibody or an isotype control, over a 6 h time course (Figure 5B). The endocytosis of 6D11 showed a progressive increase in intensity, with a significant increase in fluorescence signal already observed after 1 h of incubation (Figure 5B). In contrast, the fluorescence signal from the isotype control antibody remained consistently low over the 6 h incubation (Figure 5B). The monoclonal antibody 6D11 displayed similar punctate labelling for live LNCaP cells compared to fixed and permeabilized cells (Figure 5C,D), which was also consistent with a high level of antibody uptake into the live cells. There was evidence of Alexa Fluor^®^ 647-conjugated 6D11 monoclonal antibody delivery to EEA1 early endosomes, Rab7 late endosomes, and LAMP-1 endosomes-lysosomes after 1–4 h incubation on live LNCaP cells, indicating delivery into different endocytic compartments (Figure 6).

### 2.5. Targeting Prostate Cell Lines with MMAE-Conjugated Anti Sortilin and Syndecan-1 Antibodies

**Figure 7:**
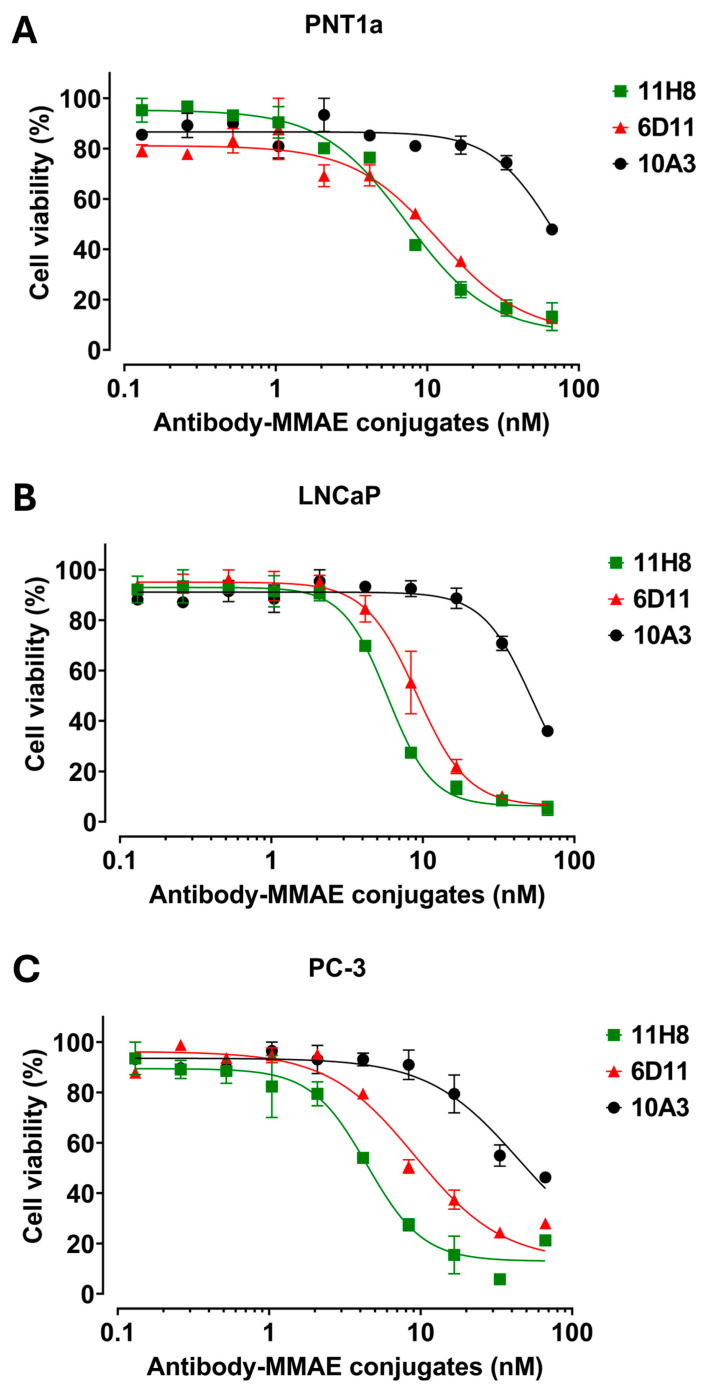
In vitro cytotoxicity of sortilin 11H8-MMAE conjugate (green), syndecan-1 6D11-MMAE conjugate (red), and isotype control 10A3-MMAE conjugate (black) in non-malignant prostate cell line PNT1a (**A**) and prostate cancer cell lines LNCaP (**B**) and PC-3 (**C**).

As a first step, MMAE was conjugated to the 11H8, 6D11 and 10A3 antibodies. The 10A3 antibody was included as a control, recognizing an internal cytosolic epitope, which is not accessible from the surface of live cells. An indirect ELISA confirmed all three MMAE-conjugated monoclonal antibodies to have equivalent binding capacity to the specific antigenic peptides as the respective non-conjugated antibodies (Supplementary Figure S1). The DAR values for the three antibody-MMAE conjugates were sortilin 11H8-MMAE (6.4), syndecan-1 6D11-MMAE (8.1), and syndecan-1 isotype control 10A3-MMAE (7.0). The incubation of sortilin 11H8-MMAE and syndecan-1 6D11-MMAE conjugates with prostate cell lines for 48 h showed increased toxicity in prostate cancer compared to non-malignant cell lines (Figure 7; Supplementary Table S1; e.g., 4.4 nM for 11H8 and 9.2 nM for 6D11 in PC3 cells compared to 44.6 nM for the isotype control). This was selective, as the isotope control reacting to the intracellular syndecan-1 epitope 10A3 had significantly less cytotoxicity; note that there was still some residual toxicity in PNT1a cells (Supplementary Table S1; 70 nm for 10A3 in PNT1a cells) that was not optimal and may indicate some Fc receptor-mediated uptake. In addition, concerns were raised about the efficacy of MMAE-induced cell death, as the 6D11 monoclonal antibody against syndecan-1 was internalized to a much greater extent than the 11H8 monoclonal antibody against sortilin in the fluorescent labelled antibody uptake experiments (Figure 5B,C for syndecan-1 and Figure 2B,C for sortilin), whereas the cytotoxicity data indicated that 11H8 had more effect than 6D11 for both LNCaP and PC-3 cells.

The cytotoxic effects of the MMAE-conjugated monoclonal antibodies were confirmed by morphological examination of prostate cells after 48 h of exposure to different concentrations of the anti-sortilin 11H8-MMAE, anti-syndecan-1 6D11-MMAE or anti-syndecan-1 10A3 MMAE (Supplementary Figures S2–S4). Anti-sortilin 11H8-MMAE induced morphological changes in the LNCaP and PC-3 malignant prostate cell lines at 8 nM (including cell shrinkage, rounding, and loss of adherence), whereas the non-malignant prostate cell line PNT1a only exhibited these changes at 33 nM. Similarly, anti-syndecan-1 6D11-MMAE induced similar morphological changes in the LNCaP and PC-3 malignant prostate cell lines at 17 nM, whereas the non-malignant prostate cell line PNT1a only exhibited these changes at 33 nM. In contrast, no morphological changes were observed in any of the prostate cell lines when exposed to the isotype control/intracellular syndecan-1 epitope reactive 10A3-MMAE at concentrations up to 33 nM.

Prostate cell lines were incubated with the antibody-MMAE conjugates for 48 h, and cytotoxicity was measured by a resazurin assay kit. The experiment was performed in duplicate for triplicate samples. The data were fitted to four-parameter logistic curves using nonlinear regression and presented as the mean ± SEM.

## 3. Discussion

Over the past two decades, the FDA has granted approval for 15 ADCs, and there are currently more than 80 ADCs undergoing clinical trials for different types of cancer [38,39]. An increasing number of studies have focused on ADCs for prostate cancer therapy, due to the promise of therapeutic efficacy observed in other malignancies [18,39]; but there are still no ADCs approved for advanced or CRPC. Recently, two cell surface integral membrane proteins, sortilin and syndecan-1, were reported to define critical aspects of prostate cancer pathogenesis in patient tissue samples [4]. Sortilin and syndecan-1 have pivotal roles in prostate cancer development and are specifically linked to different stages of cancer progression [4,27,28]. Sortilin is expressed in low-grade cancer, but is reduced during disease progression, where there are metabolic shifts in the cancer cells involving either lipid- or glucose-dependent regulation. Meanwhile, syndecan-1 expression has been correlated with a lipogenic phenotype in advanced prostate cancer [4,27,28,33,40,41,42,43,44,45] and has been linked to αvβ3 integrin activation in breast cancer [46]. Syndecan-1 upregulation in advanced prostate cancer stages may enhance disease progression and therapeutic resistance by altering the levels of αvβ3 integrin and survivin [47,48,49,50]. The combination of sortilin and syndecan-1 can be used to accurately map and interpret the grade of the cancer [4,28], giving a complete picture of the cancer biology and identifying early-stage through to late-stage cancer. Given the interplay between sortilin and syndecan-1, therapeutic approaches that can target both molecules could address the dual phases of cancer development/progression and help prevent metabolic shifts in either direction.

Here, sortilin and syndecan-1 were selected as therapeutic targets for prostate cancer due to their capacity to recognize different morphologies in cancer tissue, as well as their specific regulatory/functional roles in cell metabolism [40,41,44,51,52,53,54]. To effectively target sortilin and syndecan-1 on the cell surface of prostate cancer cells, specific anti-sortilin and anti-syndecan-1 monoclonal antibodies were generated against extracellular domains of these integral membrane proteins. One of the critical issues ADCs encounter in clinical trials is a failure to internalize into target cells [55]. We demonstrated successful target interaction and endocytosis of fluorescent labelled anti-sortilin and anti-syndecan-1 monoclonal antibodies, with notably more efficient uptake of the 6D11 anti-syndecan-1 antibody. This result is ideal for the treatment of advanced cancer where the expression of syndecan-1 is significant in high-grade cancer and is predictive of poor clinical outcomes [28]. Having demonstrated the capacity to target and internalize 11H8 anti-sortilin and 6D11 anti-syndecan-1 antibodies, attention turned towards weaponizing these antibodies with a toxic payload.

The most widely employed strategy for antibody conjugation is to firstly reduce the disulfide bonds present in the antibody to reveal a reactive thiol group, that can then undergo Michael addition chemistry with a maleimide handle conjugated to the chemical cargo of interest [18,56,57]. The MC-Val-Cit-PAB-MMAE linker was selected for this work, as it enables controlled release of the cytotoxin MMAE by endosomal cathepsin B cleavage of the protease-sensitive dipeptide sequence (Val-Cit). The toxic payload selected for this work was MMAE, which is featured in one-third of the clinically approved ADC examples to date [39]. The anti-sortilin 11H8-MMAE and anti-syndecan-1 6D11-MMAE conjugates had a significant effect on cell viability and morphology in metastatic prostate cancer cells, when compared to an isotype control 10A3-MMAE conjugated antibody directed against an intracellular epitope on syndecan-1. This finding substantiated a specific role for antibody targeting in the delivery and internalization of the ADCs into prostate cancer cells.

There were some discrepancies noted for fluorescence uptake and ADC killing. The fluorescence uptake for the 6D11 anti-sortilin monoclonal antibody was much greater than for the 11H8 anti-syndecan-1 monoclonal antibody (~3 fold over a six-hour time frame), yet the two MMAE-conjugated antibodies had similar killing capacity. In addition, while there were some differences in the IC50 values of sortilin 11H8-MMAE and syndecan-1 6D11-MMAE conjugates in malignant compared to non-malignant prostate cell lines, these were also not as large as expected based on the fluorescent uptake data. One possible explanation for this observation is that the non-malignant prostate cell line (PNT1a) may not be an ideal control, as it exhibits high expression of sortilin and syndecan-1, together with cytogenetic and partial oncogenic alterations like those observed in prostate cancer cells [58]. This may also extend into potential malignant characteristics in terms of endocytosis/ADC uptake and altered metabolic activity involving sortilin and syndecan-1 biology/expression. Therefore, future investigations should consider utilizing alternative cell line models, organoids/3D bio-printed tissue or explant cultures to provide a more clinically relevant representation of the disease manifestation. The discrepancy with cell killing could also be explained by reduced cleavage of the valine-citrulline linker, due to retention of the complex in the recycling endosomes and or poor delivery to catalytic compartments required to break the linker and release the MMAE. In addition, the DAR is a crucial property of any ADC that directly influences the toxicity of the therapeutic agent [59]. A low DAR for the antibody-MMAE conjugate may result in an insufficient antitumor therapeutic effect, while a high DAR can impact the structural integrity of the conjugate, potentially affecting its reactivity and solubility [59]. The sortilin 11H8-MMAE and syndecan-1 6D11-MMAE conjugates had high DAR values, ranging from 6 to 8. Antibody-MMAE conjugates with a value of 8 tend to exhibit increased toxicity and a reduced therapeutic index compared to those treated with a DAR value of 4 [60]. The current PSMA antibody-MMAE conjugate used clinically for prostate cancer treatment has a low DAR value of approximately 3.5 and when used in prostate cancer LNCaP cell lines, resulted in a significant difference in the IC50 value of this conjugate between the LNCaP cell line and a control cell line model [61]. Consequently, the high DAR values for the sortilin 11H8-MMAE and syndecan-1 6D11-MMAE conjugates could result in more general toxicity and explain the IC50 values in non-malignant cell lines.

A second possible explanation for the reduced toxicity of the MMAE conjugates is the altered endosome biology in prostate cancer [62,63] that may have affected the delivery or conditions for cathepsin catalysis. Cathepsin catalysis is required to release the MMAE cytotoxic compound and leads to cell cycle arrest at the G2/M phase [64]. Inefficient catalysis due to altered endosome-lysosome biogenesis may explain why the fluorescent tagged syndecan-1 antibody showed extensive uptake (compared to the sortilin antibody), yet the MMAE-conjugated antibody did not show increased toxicity. The upregulation of recycling endosomes in prostate cancer (such as RAB4 and RAB11A) [42,65] and altered trafficking and disrupted endosome biogenesis may enhance the transport of ADCs back to the plasma membrane and limit the delivery to the acidic compartments required to release the MMAE. We were only able to show limited colocalization of ADCs with EEA1 early endosomes, Rab7 late endosomes and LAMP-1 endosomes-lysosomes. This could signify that the conjugates were being delivered to other endocytic compartments (e.g., Appl1 or Rab5 positive endosomes), which may have limited the catalysis of the linker and release of the MMAE toxic payload. To overcome the challenges of delivery and catalysis that appear to have limited the toxicity of the sortilin 11H8-MMAE and syndecan-1 6D11-MMAE ADCs in prostate cancer cells, a radiolabelled ligand may be used to exploit the antibody targeting and offer an alternative approach for cancer cell killing. Alternatively, a non-cleavable maytansine derivative [66] may be utilized instead of MMAE to conjugate the antibodies.

In this study, two ADCs equipped with cytotoxic MMAE were successfully prepared to provide a proof-of-principle ADC based on sortilin and syndecan-1 for prostate cancer treatment. Improvements to the conjugation chemistry to achieve sortilin and syndecan-1 ADCs with a DAR of approximately 3.5 would likely improve the therapeutic index of these molecules. These ADCs could then be used in vitro on organoid cultures or patient-derived xenograft models to better mimic the clinical manifestation of the disease and remove the limitations with virally transformed non-malignant cells. In vivo efficacy/toxicity studies would need to be performed to determine the therapeutic index for these new ADCs and to justify clinical trials in humans. An important inclusion in these pre-clinical and clinical studies would be the assessment of potential off-target tissue toxicity. Further development of the ADCs generated here offers a new and innovative strategy to potentially address the morphological and metabolic heterogeneity of prostate cancer [67]. A therapeutic intervention that limits the adaptive potential of prostate cancer has the potential to treat patients who present with either early- or late-stage disease, or to address any potential bidirectional changes between glycolytic and lipogenic metabolic phenotypes, an outcome that would be transformative for clinical practice and patient survival.

## 4. Materials and Methods

### 4.1. Antibody Reagents

Primary antibodies were purchased from Abcam (Cambridge, UK; ab16640 rabbit anti-sortilin polyclonal; ab128936 rabbit anti-syndecan-1 monoclonal; ab109110 rabbit anti-EEA1 monoclonal; ab137029 rabbit anti-Rab7 monoclonal; and ab24170 rabbit anti-LAMP-1 polyclonal) or Cell Signaling Technology (Danvers, MA, USA; rabbit anti-GAPDH monoclonal antibody #2118). Secondary antibodies were purchased from Li-COR (Goat anti-Rabbit IgG (IRDye^®^ 680RD); and Goat anti-Mouse IgG2b-Specific (IRDye^®^ 800CW)), Invitrogen (Carlsbad, CA, USA; Donkey anti-Mouse IgG (Alexa Fluor^®^ 647); and Donkey anti-Rabbit IgG (Alexa Fluor^®^ 488)), or Sigma-Aldrich (St. Louis, MI, USA; HRP Donkey anti-Mouse IgG Antibody).

### 4.2. Design and Production of Anti-Sortilin and Anti-Syndecan-1 Monoclonal Antibodies

The specific epitopes on sortilin (LANNTHQHVFDDLRG; IgG2b antibody) and syndecan-1 (TPRPRETTQLPT; IgG2b antibody) were selected with the aid of AbDesigner [68] and Phyre2 [69], and protein (in Expi293F or *E. coli* cells) and antibody production (GLP standard production in C57BL/6 mice) outsourced to GenScript (Piscataway, NJ, USA; as described in [70]). GenScript adhered to ISO 9001 standard reagent production with full peptide and protein ELISA specificity and Western blotting characterization. The affinity of the monoclonal antibodies was determined by BiaCore analysis against the peptides above; the anti-sortilin 11H8 antibody had a Kd of 3.1 × 10^−6^ M, and the anti-syndecan-1 antibody had a Kd of 4.9 × 10^−7^ M. Alexa Fluor^®^ 647 antibody labelling kit (A20186, Life Technologies Australia Pty Ltd., Melbourne, VIC, Australia) was used to label anti-sortilin and anti-syndecan-1 monoclonal antibodies.

### 4.3. Cell Culture

Human prostate cell lines PNT1a (#95012614), LNCaP (clone FCG, #89110211), 22RV1 (#05092802) and PC-3 (#90112714) and human cervical HeLa cells (#93021013) were obtained from the European Collection of Authenticated Cell Cultures (ECACC) from CellBank Australia (Children’s Medical Research Institute, Westmead, Sydney, NSW, Australia) and cultured in RPMI 1640 medium from Gibco (Thermo Fisher Scientific Australia Pty Ltd., Melbourne, VIC, Australia), supplemented with 10% FBS at 37 °C with 5% CO_2_. All cell lines used in this study (passage number < 30) underwent authentication through short tandem repeat (STR) profiling provided by ECACC. Mycoplasma testing was performed using a Lonza mycoplasma kit (Lonza Group, Basel, Switzerland).

### 4.4. Cell Lysates

Prostate cells were harvested at a confluence of 80–90%, washed once with cold 1x DPBS and extracted in 750 μL of ice-cold RIPA buffer (Merk Millipore, Burlington, MA, USA) containing 1% (*v*/*v*) Halt™ Protease and Phosphatase Inhibitor Cocktail (100×; Thermo Fisher Scientific Australia Pty Ltd., Melbourne, VIC, Australia). Cells were harvested using a rubber cell scraper and homogenized by passing through a 26-gauge syringe needle 10 times. The lysate samples were collected after centrifuging the cell extract at 16,000× *g* for 5 min at 4 °C. Total protein concentrations of lysate samples were quantified using a bicinchoninic acid assay (Pierce™ BCA Protein Assay Kits, Thermo Fisher Scientific, Waltham, MA, USA) according to the manufacturer’s instructions and quantified using an EnSpire Multimode Plate Reader (PerkinElmer Pty Ltd., Melbourne, VIC, Australia).

### 4.5. SDS-PAGE and Western Blotting

All protein samples were heat-denatured for 5 min at 95 °C in 1x NuPAGE™ LDS Sample Buffer (4×) and 1x NuPAGE™ Sample Reducing Agent (10×; Invitrogen). Samples were separated using 8% or 10% Bolt Bis-Tris pre-cast gel in an Owl electrophoresis system (Thermo Fisher Scientific Australia Pty Ltd., Melbourne, VIC, Australia), buffered with 1x Bolt MES SDS Running Buffer (20×) (Invitrogen), then electrophoresed at 130 V (fixed, with variable current) for 1 h. Following electrophoresis, protein was transferred to nitrocellulose transfer membrane (iBlot™ 2 Transfer Stacks; Thermo Fisher Scientific) using method P0 (7 min, with variable voltage of 20 V, 23 V, and 25 V) from an iBlot 2 Dry Blotting System (Thermo Fisher Scientific Australia Pty Ltd., Melbourne, VIC, Australia). The transfer membranes were blocked for 1 h at room temperature (RT) using either 5% (*w*/*v*) skim milk in TBS or 5% (*w*/*v*) BSA in TBS. Subsequently, the membranes were incubated overnight at 4 °C with either sortilin or syndecan-1 antibodies diluted in blocking buffer and washed 3× with 0.1% (*v*/*v*) TBS-Tween^®^-20 for 5 min at RT. Secondary antibody was diluted 1:10,000 in 5% (*w*/*v*) BSA in TBS buffer and incubated with the transfer membrane for 1 h at RT with gentle agitation. The membranes were then washed 3× with 0.1% (*v*/*v*) TBS-Tween^®^-20 for 5 min each and once with deionized water. Antibody detection was performed using the Odyssey CLx Imaging System (Li-COR, Lincoln, NE, USA). GAPDH was used as a loading control and detected using a rabbit anti-GAPDH antibody. Images were quantified using Empiria Studio Software (version 1.3, Li-COR). The molecular weight of detected proteins was determined through lane-analysis using Empiria Studio Software. Expression and signal density of detected proteins were normalized to GAPDH.

### 4.6. Conditional Sortilin Knockout HeLa Cell Extract for Western Blotting

Wild type (WT) HeLa cell and sortilin knockout (KO) HeLa cell lysates were purchased from Abcam (ab257696, Abcam Australia Pty Ltd., Melbourne, VIC, Australia). Knockout was accomplished by utilizing CRISPR/Cas9 technology, resulting in a variety of genetic alterations, including a deletion of 11 base pairs in exon 5, a deletion of 16 base pairs in exon 5, a 1 base pair insertion in exon 5, and an insertion of the selection cassette in exon 5.

### 4.7. Syndecan-1 Overexpression HEK293T Cell Extract for Western Blotting

Wild-type (WT) HEK293T cell and transient syndecan-1 overexpression (OE) HEK293T cell lysates were purchased from Novus Biologicals (NBP2-10760, Novus Biologicals Pty Ltd., Centennial, CO, USA). The lysate was created in HEK293T cells, using Plasmid ID RC217847 and based on accession number NM_001006946.

### 4.8. Conditional Syndecan-1 Knockout HeLa Cell Extract for Western Blotting

Wild-type (WT) HeLa cell and syndecan-1 knockout (KO) HeLa cell lysates were purchased from Creative Biogene (CLKO-0010, Creative Biogene, New York, NY, USA). Knockout was accomplished by utilizing CRISPR/Cas9 technology, resulting in a variety of genetic alterations, including a deletion of 151 base pairs in exon 5 and a deletion of 46 base pairs in intron.

### 4.9. Antibody Internalization and Immunofluorescence

For the live cell uptake studies, PNT1a (7000 cells/cm^2^), LNCaP (7000 cells/cm^2^) and PC-3 cells (4210 cells/cm^2^) were cultured in 96-well plates for 72 h. After reaching 70% confluence, the culture medium was aspirated, and the cells were incubated with 10 μg/mL of Alexa Fluor^®^ 647-labelled sortilin or syndecan-1 antibodies for specific time points at 37 °C and 5% CO_2_. Cells were then incubated with Hoechst 33,342 fluorescent dye for 10 min at 37 °C and 5% CO_2_ according to the manufacturer’s instructions (Thermo Fisher Scientific, Australia Pty Ltd., Melbourne, VIC, Australia). To remove any excess Alexa Fluor^®^ 647-labeled primary antibody and Hoechst 33,342 fluorescent dye, the cells were washed 3× with PBS. For live cell imaging, the cells were kept in fresh culture media and observed using a Zeiss Celldiscoverer 7 (CD7) microscope (Carl Zeiss Pty. Ltd., Jena, Germany) at 37 °C and 5% CO_2_. The fluorescence intensity for each image was captured with a Zeiss CD7 and quantified using ImageJ 2.0 software (as described in [27]). A negative control was included where the samples were examined without the primary antibody.

For the endosome-lysosome tracking studies, cells were seeded into ibiTreat ibidi μ-Slide VI 0.4 (80606, ibidi GmbH, Gräfelfing, Germany) and cultured for 72 h to achieve 70% confluence. The culture medium was aspirated, and cells incubated for 0–6 h with either 10 μg/mL of Alexa Fluor^®^ 647-labelled sortilin or syndecan-1 primary antibodies for specific time points at 37 °C and 5% CO_2_ (N.B. different time course uptake observed for sortilin and syndecan-1 antibodies). The culture medium was then aspirated, and cells fixed with 4% (*v*/*v*) paraformaldehyde (PFA) and 4% (*v*/*v*) sucrose in PBS at room temperature for 12 min. After fixation, cells were washed 3× with PBS, then blocked with 5% (*w*/*v*) BSA in PBS containing 0.05% (*w*/*v*) saponin for 1.5 h at room temperature. Cells were then incubated overnight at 4 °C, with EEA1 (early endosome), Rab7 (late endosome), or LAMP-1 (lysosome) primary antibodies diluted in 5% (*w*/*v*) BSA in PBS containing 0.05% (*w*/*v*) saponin. Cells were then washed 3× with PBS and co-incubated for 1 h in the dark at RT with Alexa Fluor^®^ 488 fluorophore-conjugated anti-rabbit secondary antibody and Hoechst 33,342 fluorescent dye diluted in 5% (*w*/*v*) BSA in PBS containing 0.05% (*w*/*v*) saponin. Cells were then washed 3× with PBS and once with Milli-Q water before mounting with Fluoromount Aqueous Mounting Medium (Sigma-Aldrich Australia Pty Ltd., Sydney, NSW, Australia). Confocal images were captured using a Nikon A1R+ confocal microscope (Nikon Corporation, Tokyo, Japan) and quantified as previously described [27]. Controls with single antibodies and a negative control with secondary antibody only were included in each experiment.

### 4.10. Enzyme-Linked Immunosorbent Assay (ELISA)

A 15-mer length of sortilin, syndecan-1, metalloproteinase-20, G2/mitotic-specific cyclin-B3, alpha/beta hydrolase domain-containing protein 18, plasma protease C1 inhibitor, and homeobox protein Hox-B3 were synthesized by Mimotopes (Mimotopes Pty Ltd., Melbourne, VIC, Australia). Peptides were resuspended in 50% (*v*/*v*) acetonitrile/water. Polystyrene 96-well microplates were coated with synthesized peptides (19 μg/mL) in PBS, then incubated for 1 h at 600 rpm using an orbital plate shaker (PerkinElmer Pty Ltd., Melbourne, VIC, Australia) at RT, followed by overnight incubation at 4 °C. The plate was washed with water and then incubated with PBS containing 0.1% (*w*/*v*) sodium azide and 0.1% (*v*/*v*) Tween^®^-20. ELISA plate wells were dried for 30 min at RT and then blocked for 1 h at RT and 500 rpm using a 1% (*w*/*v*) BSA solution in PBS-Tween^®^-20 (PBST). Plates were then incubated with corresponding antibody (0.25 μg/mL) in 1% (*w*/*v*) BSA blocking buffer for 1 h at RT. After rinsing with 0.1% (*v*/*v*) PBST and washing the plate 3× with PBST, plates were incubated with donkey anti-mouse HRP-conjugated secondary antibody (1:5000; AP192P, Sigma-Aldrich Australia Pty Ltd., Sydney, NSW, Australia) before washing 3× with PBST. The TMB-ELISA substrate solution (34029; Thermo Fisher Scientific Australia Pty Ltd., Melbourne, VIC, Australia) was added to each well for 20 min; reactions were stopped using 2 M sulfuric acid, and absorbance measured at 450 nm using an EnSpire™ Multimode Plate Reader (PerkinElmer, Melbourne, VIC, Australia).

Recombinant protein-based indirect ELISA was performed by GenScript as a part of the quality control during antibody production of sortilin or syndecan-1 antibodies. Recombinant sortilin or syndecan-1 proteins at a concentration of 1 μg/mL in PBS were used to coat ELISA plates with 100 μL per well. Subsequently, 10 two-fold serial dilutions of sortilin or syndecan-1 antibody were added into the ELISA plate, and the plate probed with Peroxidase-AffiniPure Goat Anti-Mouse IgG secondary antibody. The absorbance was measured at 450 nm.

### 4.11. Statistics

Statistical analysis was performed using GraphPad Prism software (version 9.3.1, GraphPad Software, San Diego, CA, USA). For the 6 h incubation period of antibody internalization in PNT1a, LNCaP and PC-3 cells, the data are presented as the mean fluorescence intensity per cell, based on three technical replicates. For statistical purposes, we assumed homogeneity based on the fluorescence uptake results. To compare the results among the three cell lines, statistical significance was assessed using a one-way ANOVA with Dunnett’s multiple comparisons test. In this comparison, each mean was evaluated against a control mean from the PNT1a cell line. A significance level of *p* ≤ 0.05 was considered statistically significant. For comparing the internalization of sortilin or syndecan-1 antibodies with that of the isotype control antibody at different time points (LNCaP cells treated with sortilin or syndecan-1 antibodies versus isotype control antibody), the data were presented as the mean fluorescence intensity per cell, and the analysis was based on three independent experiments. Statistical significance at each time point was evaluated using an unpaired *t*-test. Differences in the internalization patterns between the different antibodies (sortilin or syndecan-1 antibodies versus the isotype control antibody) at each time point were considered significant if the resulting *p*-value was ≤0.05.

### 4.12. Synthesis of Anti-Sortilin/Syndecan-1 Antibody MMAE Conjugates

The MC-Val-Cit-PAB-MMAE linker (SET0201) was purchased from Levena Biopharma (Levena Biopharma Pty Ltd., San Diego, CA, USA). The antibody-MMAE conjugates were synthesized using a modification of a reported procedure [60]. Either anti-sortilin or anti-syndecan-1 antibodies (250 μL in PBS, 4 mg/mL) were mixed with dithiothreitol (DTT, 25 μL, 100 mM in water) at RT for 1 h to facilitate the reduction of the native disulfide bonds present on the antibodies, converting the bonds into reactive thiol groups. The reduced antibodies were isolated by size-exclusion chromatography using a PD MiniTrap™ G-25 Column (Sigma-Aldrich Australia Pty Ltd., Sydney, NSW, Australia) with PBS containing 2 mmol/L ethylenediaminetetraacetic acid (EDTA). Following centrifugal concentration, the concentration of the reduced antibodies was measured by protein absorption at 280 nm, with an IgG reference, using the NanoDrop 2000 spectrophotometer (Life Technologies Australia Pty Ltd., Melbourne, VIC, Australia). Subsequently, a molar ratio of 8-fold of maleimide-linked MMAE (10 mM solution in DMSO), referred to as MC-Val-Cit-PAB-MMAE, was added to the reduced antibodies (2 mg/mL) and incubated at RT for 6 h. The reaction mixture was then subjected to purification and buffer exchange through overnight diafiltration at 4 °C, followed by two additional diafiltration steps with PBS at 4 °C to yield the final antibody MMAE conjugate product. The resulting conjugate was then subjected to sterile filtration utilizing a 0.22 μm filter. To quantify the antibody MMAE conjugates, a bicinchoninic acid assay method was used, according to the manufacturer’s instructions (Pierce™ BCA Protein Assay Kits, Thermo Fisher Scientific Australia Pty Ltd., Melbourne, VIC, Australia).

To determine the drug–antibody ratio (DAR) of antibody MMAE conjugates, the UV absorbance of antibody MMAE conjugates at 248 nm and 280 nm was measured using a NanoDrop 2000 Spectrophotometer. DAR was calculated using the following Equation (1), as described previously [60]:

(1) $$DAR=\frac{\varepsilon_{mab}^{248}-{R\varepsilon }_{mab}^{280}}{{R\varepsilon }_{MMAE}^{280}{-\varepsilon }_{MMAE}^{248}}$$

where $\varepsilon_{\mathrm{mab}}^{248}$ and $\varepsilon_{\mathrm{mab}}^{280}$ are extinction coefficients of antibodies at 248 and 280 nm, respectively; R is the ratio of UV absorbance from 248 nm to 280 nm of each conjugate (R = A248/A280); and $\varepsilon_{\mathrm{MMAE}}^{280}$ and $\varepsilon_{\mathrm{MMAE}}^{248}$ are extinction coefficients of MMAE at 280 nm and 248 nm, respectively.

### 4.13. Determination of Antibody Reactivity of ADCs

To determine antibody reactivity of ADCs, a synthetic peptide-based indirect ELISA was used to assess the binding characteristics of each antibody MMAE conjugate to synthetic 15-mer peptide sequences corresponding to the sortilin or syndecan-1 epitopes (Mimotopes Pty Ltd., Melbourne, VIC, Australia).

### 4.14. Assessment of Cell Viability Affected by Antibody-MMAE Conjugates in Prostate Cell Lines

PNT1a, LNCaP, and PC-3 cells were seeded in 96-well plates at densities of 9400 cells/cm^2^ (for PNT1a and LNCaP) and 6300 cells/cm^2^ (for PC-3) with phenol red-free RPMI 1640 culture media containing 10% FBS. After 24 h of incubation, cells reaching 40% confluency were treated in duplicate with 2-fold serial dilutions of ADCs, spanning concentrations from 0.13 nM to 67 nM. Additionally, 200 nM free MC-Val-Cit-PAB-MMAE was used as a positive control. After a 48 h incubation, cell viability was determined using the resazurin assay kit (ab112119, Abcam Australia Pty Ltd., Melbourne, VIC, Australia) according to the manufacturer’s protocol. Briefly, the cells were incubated with resazurin solution (equivalent to 20% of cell culture volume) for 4 h at 37 °C. The alterations in resazurin fluorescence were quantified using a EnSpire™ Multimode Plate Reader (PerkinElmer Pty Ltd., Melbourne, VIC, Australia), with excitation at 540 nm and emission at 590 nm. Following background fluorescence correction, the cell viability of treated cells was normalized using the readings obtained from untreated negative control and positive control groups (200 nM free MC-Val-Cit-PAB-MMAE). The untreated negative control group was set as having 100% cell viability, while the positive control group (200 nM free MC-Val-Cit-PAB-MMAE) was established with 0% cell viability. The half maximal inhibitory concentration (IC50) values and 95% confidence intervals for each ADC were calculated by fitting the data to the four-parameter logistic equation using GraphPad Prism software (version 9.3.1, GraphPad Software, San Diego, CA, USA) for data analysis.

### 4.15. Evaluation of Morphological Alterations and Apoptosis Induction by Antibody-MMAE Conjugates in Prostate Cell Lines

PNT1a, LNCaP, and PC-3 cells were seeded in 96-well plates at densities of 9400 cells/cm^2^ (for PNT1a and LNCaP) and 6300 cells/cm^2^ (for PC-3) with phenol red-free RPMI 1640 culture media containing 10% FBS. After 24 h of incubation, the cells were treated in duplicate with 2-fold serial dilutions of ADCs, spanning concentrations from 0.4 nM to 33 nM. Additionally, 200 nM free MC-Val-Cit-PAB-MMAE was used as a positive control. After a 48 h incubation, both morphological alterations and cell apoptosis were evaluated.

Morphological alterations were examined using a Zeiss CD7 microscope and associated ZEISS ZEN 3.1 blue edition software (Carl Zeiss Pty. Ltd., Jena, Germany). Phase gradient contrast images were automatically captured from the entire well under an auto-corrective 5× Plan-Apochromat Objective (NA 0.35) at a resolution of 58,043 × 38,532 pixels. Based on image capture resolution, lens and zoom factor, each pixel was equivalent to 1.823 μm in dimension.

Cell apoptosis was determined using the ApoTox-Glo™ Triplex assay (G6320, Promega Australia, Sydney, NSW, Australia), a comprehensive assay designed to assess cell viability, cytotoxicity, and apoptosis events. The assay was conducted in accordance with the manufacturer’s protocol. Briefly, the cells were incubated with viability/cytotoxicity reagent for 2 h at 37 °C. Then, the changes in cell viability fluorescence (with excitation at 405 nm and emission at 495 nm) and cytotoxicity fluorescence (with excitation at 480 nm and emission at 530 nm) were quantified using the VICTOR Nivo Multimode Microplate Reader (PerkinElmer Pty Ltd., Melbourne, VIC, Australia). After the determination of cell viability and cytotoxicity, the cells were incubated with apoptosis detection reagent (Caspase-Glo^®^ 3/7 reagent) to measure the caspase 3/7 activity. The luminescence signals were measured after 2 h of incubation at RT using the same microplate reader mentioned above. Following background fluorescence/luminescence correction, the acquired data were fitted to the four-parameter logistic equation using GraphPad Prism software (version 9.3.1, GraphPad Software).

## Data Availability

The original contributions presented in this study are included in the article/Supplementary Material. Further inquiries can be directed to the corresponding author.

## Supplementary Materials

The following supporting information can be downloaded at: https://www.mdpi.com/article/10.3390/ijms262211145/s1.

## Abbreviations

The following abbreviations are used in this manuscript:

ADCs	Antibody-drug conjugates
MMAE	Monomethyl aurastatin E
ADT	Androgen deprivation therapy
CRPC	Castration-resistant prostate cancer
AR	Androgen receptor
PSMA	Prostate-specific membrane antigen
PSCA	Prostate stem cell antigen
GLUT4	Glucose transporter type 4
LPL	Lipoprotein lipase
NARC-1	Neural apoptosis-regulated convertase 1
LDLR	Low-density lipoprotein receptor
ECACC	European Collection of Authenticated Cell Cultures
FBS	Fetal Bovine Serum
STR	Short tandem repeat
DPBS	Dulbecco’s Phosphate Buffered Saline
RIPA	Radioimmunoprecipitation assay buffer
BCA	Bicinchoninic acid assay
LDS	Lithium dodecyl sulfate
SDS	Sodium dodecyl sulfate
RT	Room temperature
BSA	Bovine serum albumin
TBS	Tris-buffered saline
GAPDH	Glyceraldehyde-3-phosphate dehydrogenase
WT	Wild type
KO	Knock out
CRISPR	Clustered regularly interspaced short palindromic repeats
CD7	Cell discoverer 7
PFA	Paraformaldehyde
PBST	PBS-Tween^®^-20
HRP	Horse radish peroxidase
ELISA	Enzyme-linked immunosorbent assay
ANOVA	Analysis of variance
DTT	Dithiothreitol
EDTA	Ethylenediaminetetraacetic acid
DMSO	Dimethyl sulfoxide
DAR	Drug-antibody ratio
UV	Ultraviolet
IC	Inhibitory concentration
BLAST	Basic Local Alignment Search Tool
FDA	Food and Drug Administration
DAPI	4′,6-diamidino-2-phenylindole
SEM	Standard error of the mean
