# Supplementary material for: Targeting Prostate Cancer Cells Using Anti-Sortilin and Anti-Syndecan-1 Antibody Drug Conjugates
Source: Int J Mol Sci. 2025 Nov 18;26(22):11145. doi: 10.3390/ijms262211145 (PMC12652466; doi:10.3390/ijms262211145)
Supplement: Supplementary file 1 [file ijms-26-11145-s001.zip › Supplementary Figures.pdf]

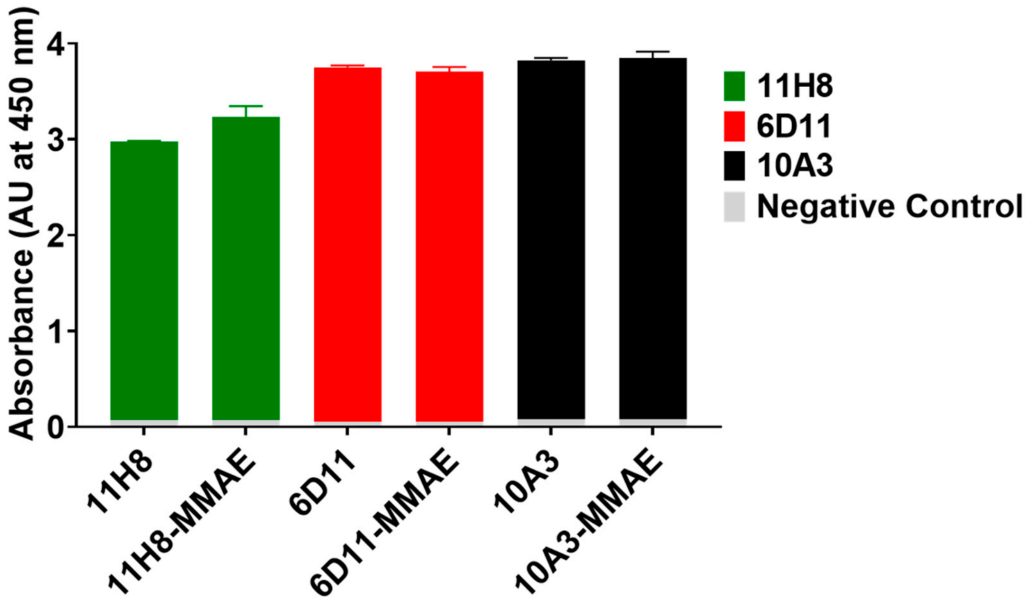

Figure S1. Characterization of monoclonal antibody-MMAE conjugate activity. Indirect ELISA confirmed that the monoclonal antibodies and MMAE conjugated anti-sortilin (11H8; LANNTHQHVFDDLRG) and anti-syndecan-1 monoclonal antibodies (6D11; ATPRPRETTQLPTTH and an isotype control antibody 10A3 recognizing an internal cytosolic epitope on syndecan-1; PKQANGGAYQKPTKQ) had equivalent reactivity with the peptide sequences for the respective epitopes. The experiment was performed in duplicate for triplicate samples. Results presented as the mean ± SEM.

Table S1. IC50 values and 95% confidence intervals for 11H8-MMAE, 6D11-MMAE and isotype control 10A3-MMAE conjugated monoclonal antibodies

Cells	Sortilin 11H8-MMAE IC_{50} (nM) (95% confidence interval)	Syndecan-1 6D11-MMAE IC_{50} (nM) (95% confidence interval)	Isotype control 10A3-MMAE IC_{50} (nM) (95% confidence interval)
PNT1a	7.3 (6.1–8.6)	11.8 (9.0–15.3)	70.0 (59.0–95.5)
LNCaP	5.8 (5.3–6.4)	9.1 (8.0–10.4)	52.5 (47.2–58.6)
PC-3	4.4 (3.5–5.5)	9.2 (7.4–11.6)	44.6 (35.9–57.5)

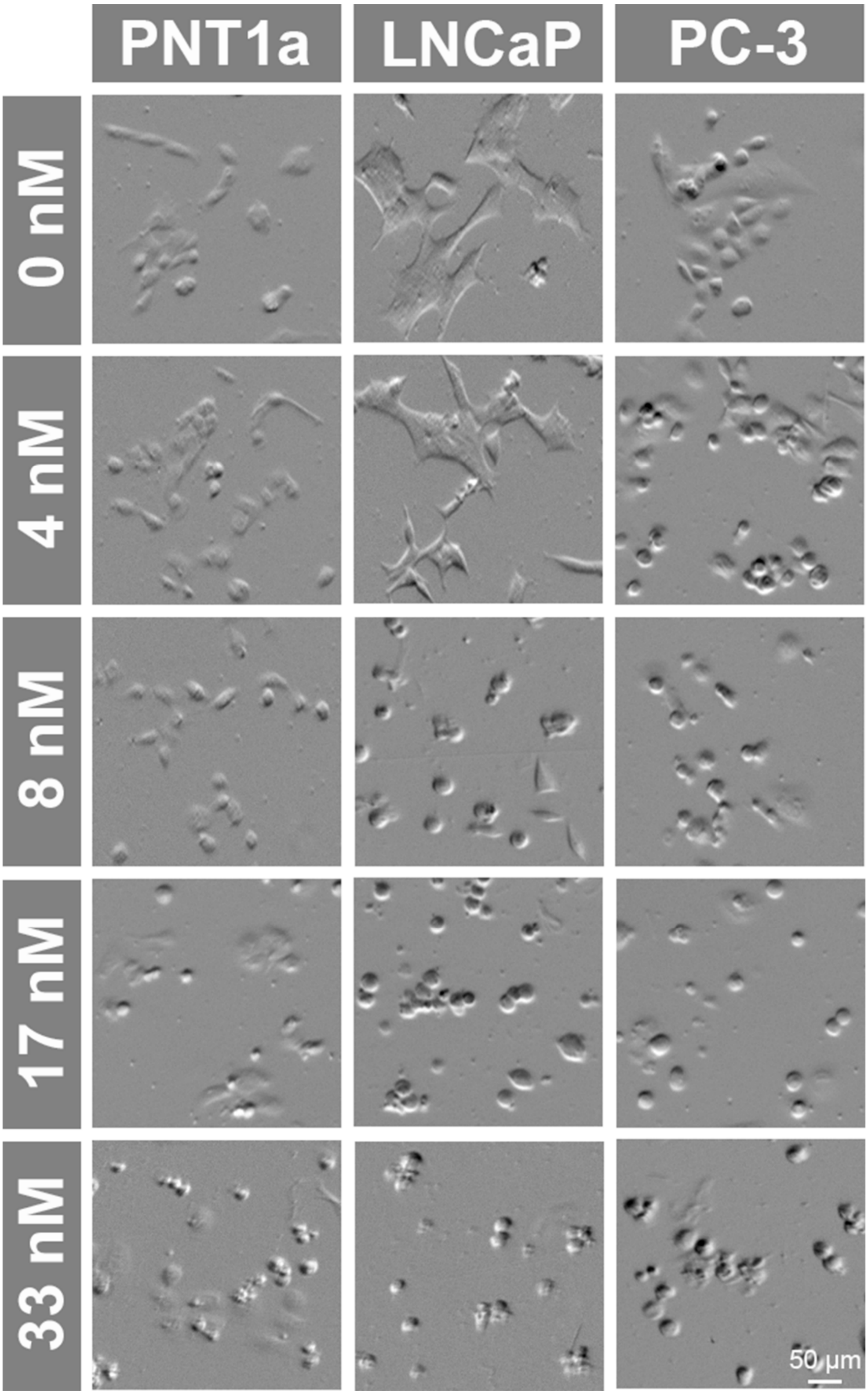

Figure S2. Morphological analysis of PNT1a non-malignant and LNCaP and PC-3 prostate cancer cell lines treated with 11H8-MMAE. Prostate cells were incubated with 0 nM to 33 nM of the MMAE conjugated antibodies for 48 hours. Representative phase contrast images were captured by Celldiscoverer 7 (Duplicate experiments were performed).

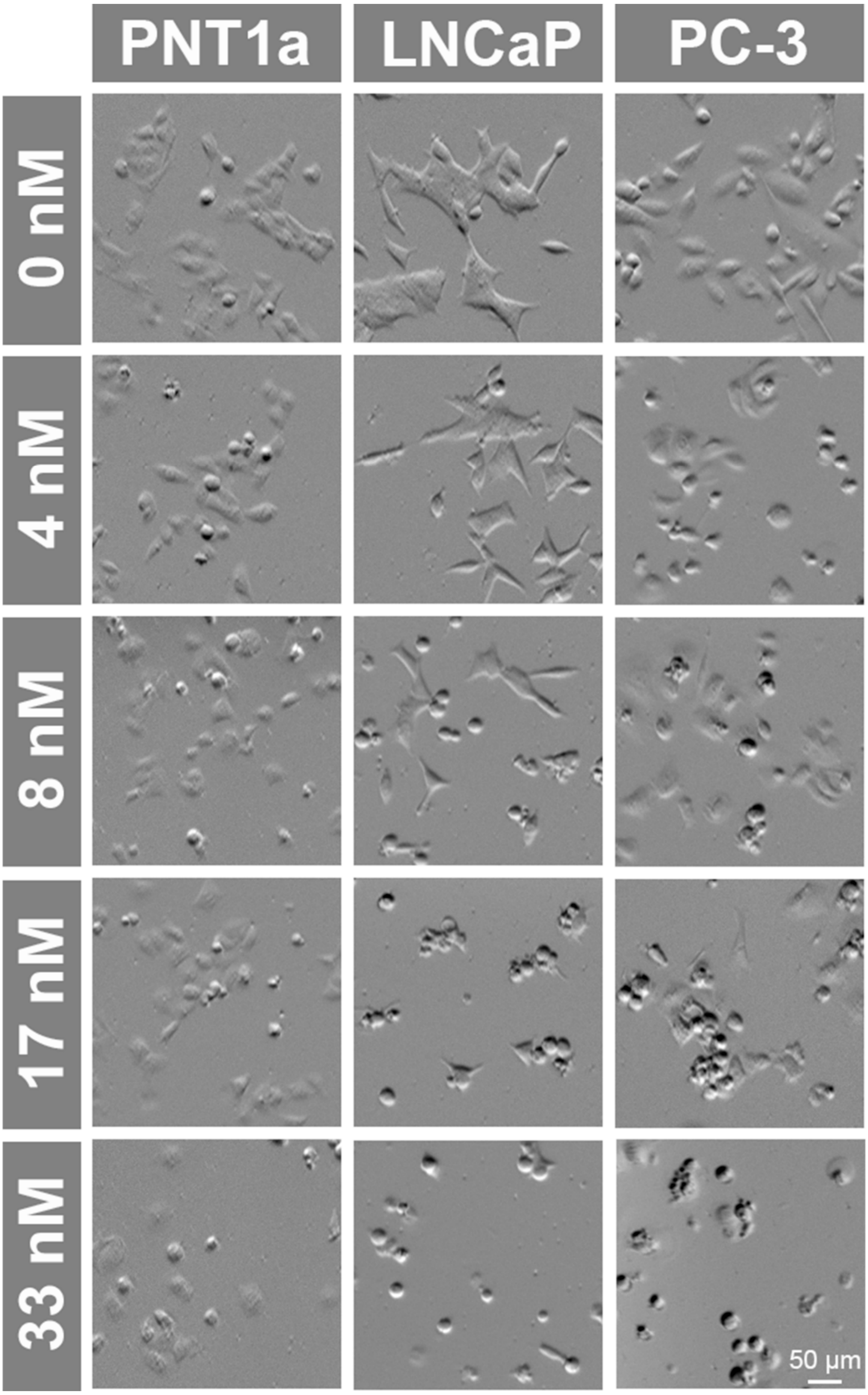

Figure S3. Morphological analysis of PNT1a non-malignant and LNCaP and PC-3 prostate cancer cell lines treated with 6D11-MMAE. Prostate cells were incubated with 0 nM to 33 nM of the MMAE conjugated antibodies for 48 hours. Representative phase contrast images were captured by Celldiscoverer 7 (Duplicate experiments were performed).

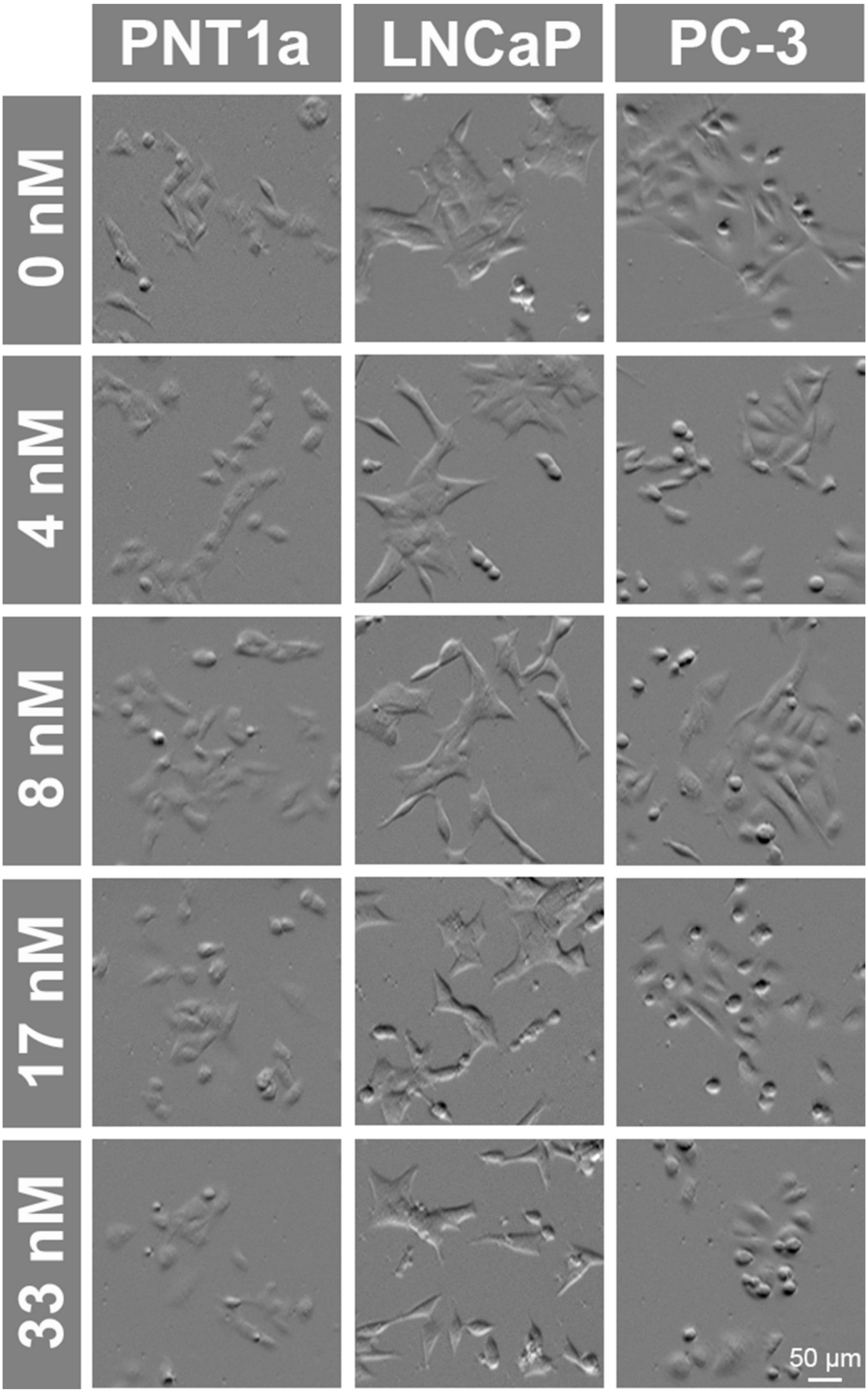

Figure S4. Morphological analysis of PNT1a non-malignant and LNCaP and PC-3 prostate cancer cell lines treated with negative control 10A3-MMAE. Prostate cells were incubated with 0 nM to 33 nM of the MMAE conjugated antibodies for 48 hours. Representative phase contrast images were captured by Celldiscoverer 7 (Duplicate experiments were performed).
